# Secreted in Xylem Genes: Drivers of Host Adaptation in *Fusarium oxysporum*

**DOI:** 10.3389/fpls.2021.628611

**Published:** 2021-04-22

**Authors:** Pooja Jangir, Namita Mehra, Karuna Sharma, Neeraja Singh, Mamta Rani, Rupam Kapoor

**Affiliations:** Department of Botany, University of Delhi, New Delhi, India

**Keywords:** *Fusarium oxysporum*, *Fusarium oxysporum* species complex, Secreted in Xylem, effectors, pathogenicity, host specificity

## Abstract

*Fusarium oxysporum* (*Fo*) is a notorious pathogen that significantly contributes to yield losses in crops of high economic status. It is responsible for vascular wilt characterized by the browning of conductive tissue, wilting, and plant death. Individual strains of *Fo* are host specific (*formae speciales*), and approximately, 150 forms have been documented so far. The pathogen secretes small effector proteins in the xylem, termed as Secreted in Xylem (Six), that contribute to its virulence. Most of these proteins contain cysteine residues in even numbers. These proteins are encoded by *SIX* genes that reside on mobile pathogenicity chromosomes. So far, 14 proteins have been reported. However, *formae speciales* vary in *SIX* protein profile and their respective gene sequence. Thus, *SIX* genes have been employed as ideal markers for pathogen identification. Acquisition of *SIX*-encoding mobile pathogenicity chromosomes by non-pathogenic lines, through horizontal transfer, results in the evolution of new virulent lines. Recently, some *SIX* genes present on these pathogenicity chromosomes have been shown to be involved in defining variation in host specificity among *formae speciales*. Along these lines, the review entails the variability (*formae speciales*, races, and vegetative compatibility groups) and evolutionary relationships among members of *F. oxysporum* species complex (FOSC). It provides updated information on the diversity, structure, regulation, and (a)virulence functions of *SIX* genes. The improved understanding of roles of *SIX* in variability and virulence of *Fo* has significant implication in establishment of molecular framework and techniques for disease management. Finally, the review identifies the gaps in current knowledge and provides insights into potential research landscapes that can be explored to strengthen the understanding of functions of *SIX* genes.

## Introduction

*Fusarium* is a complex and an adaptive genus in Ascomycota that includes both pathogenic as well as non-pathogenic species (Mandeel and Baker, 1991; Gordon and Martyn, 1997). Under this genus, species *Fusarium oxysporum* Schlechtendal (1824) emend. Snyder and Hansen (1940) (*Fo*) represents the most pervasive, anamorphic, and polytypic soil-borne pathogen (O’Donnell and Cigelnik, 1997; O’Donnell et al., 1998) that is capable of infecting more than 150 plant species. The host range of *Fo* varies from vegetables (bottle gourd and tomato), flowers (tulips and carnations), field crops (cotton and chickpea) to plantation crops (banana, dates, and palms) (Pietro et al., 2003; Rana et al., 2017; Edel-Hermann and Lecomte, 2019). Despite showing a broad host range, strains of *Fo* are highly host specific and are genetically and morphologically distinct (Mandeel et al., 2005; Leslie and Summerell, 2008; Palmero et al., 2009; Edel-Hermann and Lecomte, 2019). Together, these host-specific forms constitute a consortium referred to as *F. oxysporum* species complex (FOSC) (Edel-Hermann and Lecomte, 2019). FOSC consists of causative agents of vascular wilt, stem-, root-, and crown-rot diseases of economically imperative crops worldwide (Weimer, 1944; Olivain and Alabouvette, 1999; Michielse and Rep, 2009; Gordon, 2017; Rana et al., 2017; Edel-Hermann and Lecomte, 2019). Based on its devastating impact on crop yield, *Fo* has been positioned fifth among the top 10 economically significant phytopathogenic fungi (Dean et al., 2012).

Genome-wide analysis conducted on *Fo* has revealed a two-speed genome organization; separating genomic regions required for normal development of the pathogen from relatively fast-evolving regions required for pathogenesis (Croll and McDonald, 2012; Raffaele and Kamoun, 2012; Dong et al., 2015; Fokkens et al., 2018). The host range and specificity of *Fo* are dictated by genes located on pathogenicity-associated genomic regions (Ma et al., 2010, 2015; Rep and Kistler, 2010; Williams et al., 2016). These pathogenicity-associated genes encode effector proteins, transcription factors (TFs), secreted enzymes, and proteins involved in secondary metabolism and signal transduction (Rep et al., 2002, 2004; Houterman et al., 2007; Van Der Does et al., 2008; Ma et al., 2010; Schmidt et al., 2013). Effector proteins either effectuate a compatible (virulence) response or, on interaction with their corresponding resistance (*R*) genes, result in incompatible (avirulence) reaction (Flor, 1971; Jones and Dangl, 2006). The horizontal transfer of host-specificity genes to otherwise genetically distinct lineages result in the rapid emergence of new pathogenic lines with a wider host range (Ma et al., 2010). Considering that non-pathogenic strains of *Fo* can colonize asymptomatic plants as endophytes (Kuldau and Yates, 2000), the potential of these strains to evolve into new virulent lines is a matter of major concern (Gordon and Martyn, 1997; Recorbet et al., 2003; Michielse and Rep, 2009). It is due to the evolution of new pathogens that management strategies for Fusarium wilt have not seen much success. On that account, a thorough understanding of the molecular basis of virulence in *Fo* is of primary importance as it will provide impetus to the development of efficient and effective disease control strategies. After providing an overview on the biology and variability of *Fo* pathogens, this review will focus, in particular, on Secreted in Xylem (*SIX*) genes, their diversity across *formae speciales*, their role in virulence and host specificity, and evolutionary relationships among *Fo* pathogens to better understand host–pathogen interactions and rapid emergence of new pathogenic strains.

## Host–Pathogen Interaction

**FIGURE 1:**
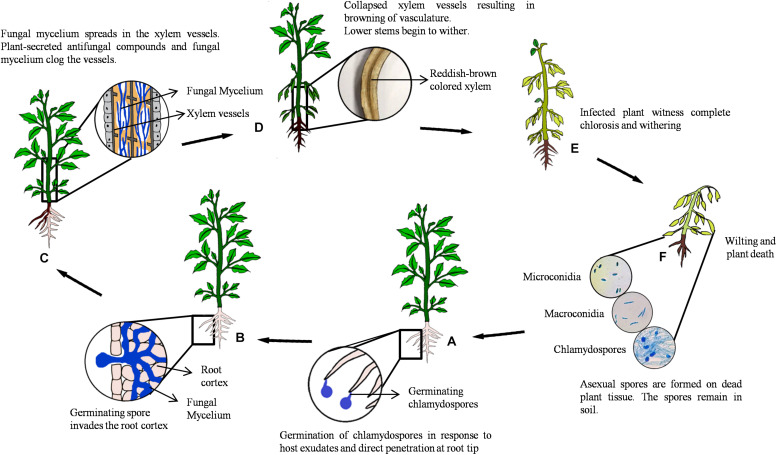
Disease cycle of *Fusarium oxysporum*. **(A)** Secretion of root exudates by host plant triggers spore germination and the development of infection hypha prompting penetration of the root epidermis at the tip. **(B)** The hypha progresses intercellularly via the root cortical cells until it enters the xylem tissue, parenchymal cells, and vessels, through xylem pits. **(C)** The pathogen colonizes vascular vessels causing blockage and browning as a result of excessive mycelial growth. **(D)** The initial stage of infection shows symptoms at the stem base and slowly advancing upward, triggering withering of young leaves. **(E)** Marginal yellowing or complete chlorosis in mature leaves is observed. **(F)** Disease progression results in wilting and death of the host plant. Fungal spores (microconidia, macroconidia, and chlamydospores) are formed on dead plant tissue and remain dispersed in soil.

Fusarium wilt is a soil-borne disease that is characterized by wilted plants with yellow leaves and a marked reduction in crop yield. The pathogen thrives in warm climate and dry soil; hence, symptoms are severe at 25–30°C (Zitter, 1998; Joshi, 2018). *Fusarium*, being an anamorphic fungus, produces asexual spores, namely, microconidia, macroconidia, and chlamydospores (dormant propagules) (Gordon, 2017). Germination of these spores is triggered by secretion of exudates from host plant roots and sites of lateral root emergence or injury. Upon germination, the development of infection hypha is initiated that penetrates the root epidermis at the tip (Bishop and Cooper, 1983). Thereafter, the hypha progresses intercellularly via root cortical cells until it enters the xylem tissue. Upon reaching the vascular tissue, the fungus branches profusely and produces microconidia and macroconidia that are transported acropetally by the transpirational pull of plant system (Bishop and Cooper, 1983). Microconidia germinate, and the hyphae spread systemically throughout the host. However, contrasting results were obtained in a study by Michielse and Rep (2009), wherein neither conidiophores nor microconidia were observed in xylem vessels of infected tomato and *Arabidopsis* plants. These results did not align with the conventional idea that microconidia play an important role in colonization (Beckman, 1987). Generally, to prevent the spread of fungus, resistant plants produce antifungal compounds and occlude the lumen of the xylem vessel by tyloses (VanderMolen et al., 1987; Zhang et al., 1993). This response in susceptible hosts is generally delayed till later stages of infection. Blockage of xylem vessels eventually results in browning of the vascular tissue, a prominent symptom of Fusarium wilt. Disease progression over time leads to leaf bending, chlorosis, wilting, and eventual death of the host (Figure 1). At this stage, the fungus sporulates extensively on the surface of dead plant tissues. The disease spread to other hosts via infected plant parts, transplants or seeds, and contaminated soil (Bishop and Cooper, 1983; Michielse and Rep, 2009; Gordon, 2017; Joshi, 2018).

## Concept of *Formae speciales*, Races, and Vegetative Compatibility Groups

Pathogenic *Fusarium* isolates are differentiated at subspecies level into assemblages termed as *formae speciales* (ff. spp.) (Gordon, 1965; Armstrong and Armstrong, 1981; Baayen, 2000). A *forma specialis* (f. sp.) is composed of isolates capable of infecting a unique host. Individuals from a *forma specialis* are further subdivided into pathogenic races depending upon their varied virulence toward cultivars of the same host (Correll, 1991). New races within a *forma specialis* emerge as a result of mutations in pathogenicity-associated genes. For instance, in race 1 of *F. oxysporum* f. sp. *lycopersici (Fol)*, whose isolates express three effector genes (*AVR1*, *AVR2*, and *AVR3*), deletion of *AVR1* resulted in emergence of race 2 and point mutation(s) in *AVR2* eventuated in the evolution of race 3 (Houterman et al., 2008, 2009; Takken and Rep, 2010; Biju et al., 2017). Based on the capability of the isolates to undergo heterokaryosis, they can be grouped as vegetative compatibility groups (VCGs) (Puhalla, 1985; Ploetz and Correll, 1988; Moore et al., 1993). The members of a particular VCG are clonal lineages and share similar pathological, physiological, and biological attributes (Caten and Jinks, 1966). The relationship between races and VCGs of a *forma specialis* varies from simple to relatively complex (Correll, 1991). In a rather simple relationship, isolates from one race (even from diverse geographical backgrounds) may correspond to a single VCG. For example, in *F. oxysporum* f. sp. *niveum* (*Fon*), all race 2 isolates belong to a single VCG (Correll, 1991; Epstein et al., 2017). On the other hand, occurrence of isolates of different races within a single VCG or isolates of a single race belonging to different VCGs may add to the complexity of the relationship thereof. For instance, three VCGs of *F. oxysporum* f. sp. *cubense* (*Focub*) (0124, 0125, and 0128) contain isolates of races 1 and 2, while isolates of race 1 of *Focub* belong to eight different VCGs (0123, 0124, 0125, 0128, 01210, 01217, 01218, and 01220) (Czislowski et al., 2018).

## Polyphyletic Origin of *Fusarium oxysporum* Species Complex Members

Recent evolutionary studies have annulled the classical concept that most of the pathogenic isolates of *Fo* are monophyletic in origin. It is now well established that most *formae speciales* have evolved independently multiple times throughout the course of evolution pointing towards their para- or polyphyletic origin (O’Donnell et al., 1998, 1999). Remarkably, isolates from one *forma specialis*, race, or VCG may show close relatedness to the isolates of other *formae speciales*, races, or VCGs than their own members (Kistler, 1997; Lievens et al., 2009a). Conserved gene sequences and their combinations, mitochondrial or nuclear barcoding, and pathogenesis-related genes have been used to study the evolutionary relationships between different *formae speciales*, races, and VCGs. Studies based on IGS (intergenic spacer) region, vegetative compatibility, restriction fragment length polymorphism (RFLP), mitochondrial DNA (mtDNA) and isozyme polymorphism have demonstrated that *Fol* isolates may reflect genetically different evolutionary lines. For instance, phylogenetic analysis of *Fol* isolates by utilizing IGS rDNA sequences showed three well-supported clusters (A1, A2, and A3) (Kawabe et al., 2005). The major cluster A2 consisted of *Fol* isolates along with representatives of other *formae speciales* (*melonis*, *batatas*, and *radicis-lycopersici*). Lievens et al. (2009b) studied the evolutionary relationships between *Fol* and *F. oxysporum* f. sp. *radicis-lycopersici* (*Forl*) by constructing phylogenetic tree employing pgx4 (exo-polygalacturonase) and Translation Elongation Factor 1α (*TEF-1*α) gene data and concluded that *Fol* comprises of three independent clonal lineages. Phylogenetic tree developed by exploiting data from the IGS region of rDNA resolved the isolates of *Fol* and *Forl* into five distinct lineages (Cai et al., 2003). Interestingly, *Fol* VCG 0035 (lineage 5) isolates had more similarities to *Forl* isolates (lineage 4) compared with the isolates in other *Fol* lineages or VCGs (Cai et al., 2003). About a decade later, Nirmaladevi et al. (2016), through the ITS (internal transcribed spacer) region analysis, identified evolutionary relationships among *Fol* isolates and other *formae speciales* and concluded that *Fol* represents a polyphyletic *forma specialis* due to divergent evolution. A similar inference was drawn by employing mitochondrial small subunit (*mtSSU*) *rDNA* and *TEF-1*α-based studies in *forma specialis cubense*. These phylogenetic studies showed that *Focub* consists of four clades containing members from various polytypic species (O’Donnell et al., 1998). The results demonstrated a close relatedness of *Focub* isolates to other representative members of FOSC. Phylogenetic relationships between *Focub* and FOSC members, as well as between VCGs and *Focub* races, have revealed that the capacity of pathogens to trigger banana disease has evolved independently multiple times (Fourie et al., 2009). The ability of *Focub* to inflict disease on a particular cultivar of banana is a polyphyletic trait (Fourie et al., 2009). Multiple gene-genealogical studies established *F. oxysporum* f. sp. *vasinfectum* (*Fov*) as a polyphyletic *forma specialis* (Skovgaard et al., 2001). The phylogenetic tree obtained from integrated *TEF-1*α, *NIR* (nitrate reductase), *PHO* (acid phosphatase), and *mtSSU rDNA* sequences reported four different lineages of *Fov* that correlated with variations in their origin and virulence. The phylogenetic relationship deduced from *TEF-1*α data of *Phoenix*-specific *F. oxysporum* f. sp. *canariensis* (*Focan*) isolates reported the presence of three lineages, confirming that *Focan* in Australia evolved independently (Laurence et al., 2015). Depending on *TEF-1*α phylogenies, isolates belonging to *F. oxysporum* f. sp. *cucumerinum* (*Foc*) and *F. oxysporum* f. sp. *radicis-cucumerinum* (*Forc*) were reported as genetically diverse and resolved in clades separate from other non-cucurbit-infecting *formae speciales* (Lievens et al., 2007). Based on the phylogenetic tree obtained from 10 conserved gene dataset, Epstein et al. (2017) described *F. oxysporum* f. sp. *apii* as a polyphyletic *forma specialis*. Evaluation of genetic diversity among *forma specialis betae* isolates based on *ITS*, β-tubulin, and *TEF-1*α phylogenies reported the polyphyletic origin of this *forma specialis* (Hill et al., 2011). Isolates from *F. oxysporum* f. sp. *melonis* (*Fom*) were also reported to be polyphyletic based on the phylogenetic tree constructed using nuclear repetitive DNA sequences. The isolates were separated into different groups in the phylogenetic tree (Namiki et al., 1994; Gordon and Martyn, 1997). The *mtSSU rRNA* and *TEF-1*α phylogenies clustered *F. oxysporum* f. sp. *vanillae* isolates into different clades pointing toward a polyphyletic pattern of origin (Pinaria et al., 2015). Similarly, *F. oxysporum* f. sp. *lactucae* was described as polyphyletic based on *IGS* phylogeny and VCGs (Ogiso et al., 2002; Fujinaga et al., 2005; Pasquali et al., 2005).

Not all *formae speciales* are polyphyletic; a few monophyletic ones have also been reported. In a study by Baayen et al. (2000), *TEF-1*α, *mtSSU rDNA*, and amplified fragment length polymorphism (AFLP)-based phylogenies were assessed to identify the nature of origin in 89 isolates belonging to eight different *formae speciales*. The study revealed two *formae speciales*, *tulipae* and *lilii*, to be monophyletic and the remaining ones, *asparagi*, *dianthi*, *gladioli*, *lini*, *opuntiarum*, and *spinaciae*, to be polyphyletic in origin. Apart from *lilii* and *tulipae* (Baayen et al., 2000), *ciceris* is also considered as a monophyletic *forma specialis* (Jiménez-Gasco et al., 2002). Isolates of different *F. oxysporum* f. sp. *ciceris* (*Focic*) races shared similar sequences in the intronic region of *TEF-1*α, β-tubulin, calmodulin, actin, and histone 3 genes by virtue of which they clustered together, separated from other non-pathogenic isolates and *formae speciales* suggesting a monophyletic origin of *Focic* (Jiménez-Gasco et al., 2002). Identifying species boundary in FOSC is undeniably a challenge considering the lack of distinct morphological characters, ecological diversity, diverse genetic background, and dynamic host range of strains. FOSC members are devoid of sexual stages in their life cycle; however, horizontal gene transfer (HGT) within the complex may contribute to the observed genetic diversity.

## Features of *Fusarium oxysporum* Genome

The genome sequences of 16 species (11 *Fo* and five *Fusarium* species) can be retrieved from the Joint Genome Institute (JGI) MycoCosm site, FungiDb (Fungi database), and GenBank National Centre for Biotechnology Information (NCBI) database (Ma et al., 2010, 2014; Ma L. J. et al., 2013; Williams et al., 2016; DeIulio et al., 2018). The complete genetic and physical maps of the pathogens provide an outstanding opportunity to investigate the variation in genome size and content within the genus (Ma et al., 2010; Ma L. J. et al., 2013). Comparative genomic studies among the members of the genus have provided insight on the variation in genome size within the genus; *Fol* strain *4287* (*Fol-4287*) has an average genome size of 61 Mb, whereas *Fusarium verticillioides* (*Fv*), *Fusarium graminearum* (*Fg*), and *Fusarium solani* (*Fs*) (syn. *Nectria haematococca*) have 42-, 51-, and 36-Mb genome sizes, respectively (Ma et al., 2010). *Fv* and *Fg* have comparable genome sizes, even though in the course of evolutionary diversification, *Fol* and *Fv* lineages share a common ancestor and have diverged earlier from the clade containing the *Fg* lineage (Ma L. J. et al., 2013; O’Donnell et al., 2013). Furthermore, genome sequencing and gene mapping of *Fusarium* species have revealed a variable chromosome count, fluctuating between four in *Fg* and 17 in *Fs* (Cuomo et al., 2007; Coleman et al., 2009; Ma et al., 2010).

The *F. oxysporum* genome is compartmentalized structurally and functionally into two components: a core genome that encodes housekeeping genes vital for survival and growth of the pathogen and an accessory genome encoding pathogenicity or virulence-associated genes (Ma et al., 2010; Croll and McDonald, 2012; Schmidt et al., 2013). Till date, the genome of *Fol-4287* remains the most exhaustively studied genome that has been mapped into complete chromosome sequences. Therefore, the *Fol-4287* strain is used as a key point of reference for subsequent studies. Out of 15 chromosomes mapped in the genome assembly of *Fol-4287*, 11 are designated as core chromosomes and four as accessory chromosomes. The core genome of *Fol-4287* shows 80 and 90% similarity to *Fg* and *Fv* genomes, respectively, suggesting that they are highly syntenic across isolates and related species (Ma et al., 2010).

The accessory genome, also termed as conditionally dispensable (CD) chromosomes, supernumerary (SP) chromosomes, or lineage-specific (LS) region, encompasses 19 Mb of the total genome size and includes chromosomes 3, 6, 14, 15, scaffold 27 of core chromosome 1, and scaffold 31 of core chromosome 2 (Ma et al., 2010). Lineage-specific regions are rich in retro-elements including SINEs (short interspersed repeat elements), LINEs (long interspersed repeat elements), gypsy- and copia-like long terminal repeat retrotransposons, and DNA transposons [miniature inverted transposable elements (MITEs), hAT-like, Tc1-mariner, and Mutator-like] (Ma et al., 2010). The LS region contains 95% of DNA transposons and 74% of all transposable elements (TEs) present in the *Fol-4287* genome (Ma et al., 2010) and may be specifically associated with pathogenic adaptation (Ma et al., 2010, 2015). The shared genomic region of *Fol* and *Arabidopsis*-infecting strain (*Fo-5176*) (55 Mb) amounts to less than 2% of sequence divergence. Intriguingly, counterparts of most of the *Fol* LS region are missing in *Fo-5176* (Ma et al., 2010). Similarly, *Fov* also shows high sequence identity only to the core genomic region of *Fol* and not to the corresponding LS region (Ma et al., 2010). On this account, comparison among the genomes of *Fo* pathogens link LS regions to host adaptation (Ma et al., 2010, 2015).

**FIGURE 2:**
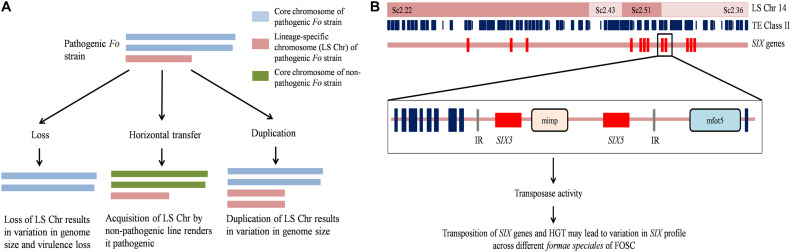
Schematic representation of *Fusarium oxysporum* genome features. **(A)** Horizontal transfer, deletion, and duplication of the lineage-specific (LS) chromosome 14 (LS Chr14) alter pathogenicity and genome size. **(B)** The LS Chr14 structure showing the presence of class II transposable elements (TE class II) and Secreted in Xylem (*SIX*) genes. TE class II elements present in the promoter region of *SIX* are miniature impala (mimp) and mfot5 elements. *SIX* genes might be trapped between the internal resolution (IR) sites of TEs and subsequently transposed together. The mobilization of *SIX* genes due to the activity of TEs and HGT could result in variation in *SIX* profile in FOSC members. *Fo*, *Fusarium oxysporum*; FOSC, *F. oxysporum* species complex; HGT, Horizontal gene transfer.

The observed variation in genome size in the genus is attributed to the activity of TEs, horizontal chromosome transfer (HCT), and deletion or fusion of genomic regions (Kistler et al., 1995; Ma et al., 2010; Ma L. J. et al., 2013; Schmidt et al., 2013) (schematically represented in Figure 2). The activity of TEs can cause translocation, deletion, and complex arrangements of genetic material (Davière et al., 2001; Schmidt et al., 2013). The increase in genome size of *Fol* has been attributed to the acquisition of LS chromosomes from other *Fusarium* species through horizontal transfer (Ma et al., 2010).

## Horizontal Transfer of Mobile Pathogenicity Chromosome

Horizontal transfer provides a mechanism to transfer pathogenicity-associated genes/chromosomes from a pathogenic isolate to a non-pathogenic one, resulting in the generation of a new virulent lineage. Along these lines, the ability of *Fol* to cause disease on tomato has been presumed to be acquired through horizontal transfer of pathogenicity chromosome from other *Fusarium* species (Ma et al., 2010). This was experimentally demonstrated via co-incubation studies. Chromosome 14 of *Fol* pathogenic strain (*Fol-007*) marked with zeocin gene was co-incubated with a non-pathogenic *Fo* strain (*Fo-47*) labeled with the hygromycin gene. Transfer of chromosome 14 during this co-cultivation experiment rendered *Fo-47* pathogenic to tomato (Ma et al., 2010). Similar outcomes in co-incubation experiments were obtained where LS chromosome were transferred from a pathogenic line (*Fol-4287*) to a non-pathogenic line that rendered it pathogenic (Vlaardingerbroek et al., 2016a). Studies have also reported HCT in *formae speciales* other than *Fol*. van Dam et al. (2017) through co-cultivation experiments assessed HCT between cucurbit-infecting strains, in which *Forc-016* was chosen as the donor strain and *Fo-47* as the recipient. All strains obtained from the experiment exhibited the karyotype of *Fo-47* strain along with an additional chromosome presumed to have been transferred from *Forc-016* as a result of HCT. Besides the members of FOSC, HGT between other *Fusarium* species and *Fo* was also reported (van Dam et al., 2017). Flower bulb-infecting strains, *Fusarium proliferatum*, *Fusarium hostae*, and *Fusarium agapanthi*, showed the presence of *Fo*-specific genes providing evidence for interspecific horizontal transfer due to shared habitat between their ancestors and *F. oxysporum* f. sp. *hyacinthi* or *F. oxysporum* f. sp. *lilii* strains. On the other hand, complete loss of chromosome 14 in pathogenic *Fol-4287* strains compromised virulence on host plants (Ma et al., 2010), whereas no effect on pathogenicity was observed on the deletion of core chromosome (Vlaardingerbroek et al., 2016b). Contrarily, strains with partial deletion of *Fol-4287* chromosome 14 regions including effector genes were still pathogenic, implicating that loss of individual or a few genes results in only fractional loss of virulence (Ma et al., 2010). Vlaardingerbroek et al. (2016b) further elaborated on these results. They showed that a part of the short arm (p arm) of the pathogenicity chromosome is adequate for inflicting disease on plants. Transfer of this portion of pathogenicity chromosome is sufficient to convert a non-pathogenic line to a pathogenic line. Interestingly, recipient strains of this portion of chromosome (short arm) were reported to be more virulent than strains that received complete pathogenicity chromosome (short and long arm). This suggested that the sequences present on the long arm (q arm) of the chromosome were possibly involved in suppressing virulence in non-pathogenic strains that received the complete chromosome (Vlaardingerbroek et al., 2016b). It was evident from these studies that LS chromosomes are significant for the development of new pathogenic lines. Owing to the limited availability of whole genome sequences of many *formae speciales*, it is difficult to trace the path of HCT between strains. More studies are needed to generate a curated database of genome assembly of pathogenic as well as non-pathogenic isolates. Analyses of the genome of isolates from different geographical backgrounds will shed light on how new pathogens evolve on the acquisition of mobile pathogenicity chromosomes from other lineages.

## Pathogenicity Factors

Xylem-colonizing *Fusarium* pathogens employ both general and specific pathogenicity mechanisms to invade the host. While components of cell signaling pathways, such as cyclic adenosine monophosphate (cAMP), mitogen-activated protein kinase (MAPK), Ras (retrovirus-associated DNA sequences) proteins, G (guanine nucleotide-binding) protein, and cell wall-degrading enzymes, encompass the general factors regulating pathogenicity (Di Pietro et al., 2001; Jain et al., 2002, 2003, 2005; Ma L. J. et al., 2013; Guo et al., 2016; Liu et al., 2016), effectors and host-specific toxins attribute specificity to pathogens. Effectors secreted by the pathogen facilitate its colonization by modulating immune response in the host plant (Hogenhout et al., 2009). Secreted in Xylem (Six) proteins is one such example of effectors (Rep et al., 2002, 2004) whose detailed overview, structure, regulation, and diverse roles are dealt in further sections.

### Secreted in Xylem Proteins

Rep et al. (2002) identified a small 12-kDa cysteine-rich fungal protein in the xylem sap proteome of tomato plants infected with *Fol*. Further structural analysis revealed that the observed 12-kDa protein corresponded to the central part (six of the eight cysteine residues) of the actual 30-kDa protein that they termed as Six1 (Rep et al., 2004). Later, Houterman et al. (2007) identified a 22-kDa propeptide of Six 1 protein along with three new Six proteins, namely, Six2, Six3, and Six4, that were approximately 24, 16, and 24 kDa in size, respectively, with eight, two, and six cysteine residues, respectively (Houterman et al., 2007). Van Der Does et al. (2008) and Schmidt et al. (2013), through genomic analysis, identified genes that encode Six5, Six6, and Six7; and Six8, Six9, Six10, Six11, Six12, Six13, and Six14 proteins, respectively. Hitherto, 14 Six proteins have been recognized in *Fol*. These are small secreted proteins, and most of them contain cysteine residues in even numbers (Rep et al., 2004; Rep, 2005; Houterman et al., 2007; Ma et al., 2010). Initially, *SIX* genes were considered to be limited to *Fol*, but later, homologs were identified in other *formae speciales* as well (Supplementary Table 1). It is noteworthy to mention that non-pathogenic strains of *Fo* share a set of conserved putative effector genes with the pathogenic strains but carry fewer *SIX* genes (van Dam et al., 2016; de Lamo and Takken, 2020).

### Structure and Regulation of Secreted in Xylem Gene

The accessory chromosome 14 of *Fol-4287* strain is dominated by TEs and has been predicted to predominantly harbor all 14 *SIX* genes (Ma et al., 2010). The presence of TEs in the genome was also associated with clustering of *SIX* genes observed in TE-rich regions. Occasionally, *SIX* genes present in the vicinity of IR (inverted repeats) sites of class II TEs might get trapped and translocated together to a new location within class II TE-rich chromosomal subregions (Schmidt et al., 2013). In accordance to that, the highly dynamic genomic location of *AVR-Pita* in rice blast fungus *Magnaporthe oryzae* was also attributed to the activity of TEs. Transposon insertion in *AVR-Pita* gene prevented the host from recognizing this avirulence protein (Zhou et al., 2007). Multiple translocation events of *AVR-Pita* resulted in a cycle of loss and gain of recognition by resistant rice cultivars (Chuma et al., 2011). Similarly, in *Fol*, deletion events caused by recombination between TEs led to the loss of an *Fol* avirulence gene (*AVR1*) that eventuated in overcoming of resistance mediated by the cognate resistance gene (Biju et al., 2017). Owing to the high density of TEs in LS regions of *Fo* pathogens, FOSC can prove useful as a model system to decipher relationships between virulence and TEs.

Structurally, *SIX* genes harbor two MITEs, namely, mimp (miniature impala; sized ≈220 nucleotides) and mFot5 (miniature Fot5 transposon) that vary in their distribution. While mfot5 has been reported to be present downstream of *SIX9* or some mini-effector clusters, a portion of mimp is found consistently present in the promoter region of all *SIX* genes (Schmidt et al., 2013). Hence, mimp can be exploited as a diagnostic feature in the detection of putative *SIX* genes. Overall, 103 mimp elements have been reported in *Fol*-4287 genome. Among these, only four are present on core chromosomes, while 54 are located on accessory chromosome 14, and the remaining 45 are on other accessory chromosomes (Schmidt et al., 2013). Homologs of five *SIX* genes (*SIX1*, *SIX2*, *SIX6*, *SIX7*, and *SIX11*) and an avirulence gene (*FomAVR2*) were identified using mimp elements in melon-*Fom* pathosystem (van Dam and Rep, 2017). Similarly, mimp elements were utilized to predict effector candidates in *Fol* (Schmidt et al., 2013), legume-infecting strains such as *Focic* and *F. oxysporum* f. sp. *pisi* (Williams et al., 2016), *F. oxysporum* f. sp. *cepae* (Armitage et al., 2018), and race 1 and 4 of *Focub* (Chang et al., 2020). Interestingly, deletion of mimp element from the promoter region of *SIX* genes (*SIX1*, *SIX3*, and *SIX5*) neither altered gene expression nor affected pathogenicity of *Fol* ruling out the direct involvement of mimp in *SIX* gene expression (Schmidt et al., 2013).

Virulence in *Fo* is considered a polygenic trait and requires TFs for the regulation of pathogenicity-related genes (Husaini et al., 2018). The role of a TF Six gene expression 1 (*SGE1*) (situated on the core genome), in modulating the expression of *SIX* genes (*SIX1*, *SIX2*, *SIX3*, and *SIX5*) has been confirmed in a study by Michielse et al. (2009b) suggesting the dependency of *SIX* expression on the core chromosome. In compliance with its transcriptional role, deletion of *SGE1* in tomato-infecting *Fol* resulted in reduced pathogenicity, which is attributable to the lost expression of effector genes. *SGE1* deletion mutants of *Fol* also exhibited a quantitative reduction in conidiation, confirming the major role of *SGE1* during parasitic growth of the pathogen (Michielse et al., 2009b). Various orthologs of *SGE1* have been reported in fungi such as *Fv* and *Candida albicans* (Michielse et al., 2009a; Brown et al., 2014). The retention of this gene in *Fol* indicates that it is a conserved TF that has developed as a *SIX* gene regulator (Michielse et al., 2009b).

Two TFs, Fusarium transcription factors (*FTF*) *1* and *2*, belonging to a Zn(II)2Cys6-type family factors, modulate the expression of *SGE1* and *SIX* genes (Niño-Sánchez et al., 2016; Van Der Does et al., 2016). While multiple copies of *FTF1* are present on chromosome 14 of *Fol-4287* and virulent strains of *F. oxysporum* f. sp. *phaseoli* (*Foph*), a single copy of *FTF2* is present in all filamentous Ascomycetes (de Vega-Bartol et al., 2011; Niño-Sánchez et al., 2015, 2016; Van Der Does et al., 2016). Studies on *SGE1* reported that the expression of *SIX* genes is dependent on the core chromosome, but the presence of *FTF* on the pathogenicity chromosome suggested that the *SIX* gene expression may also be controlled by the chromosome itself (Michielse et al., 2009b; Schmidt et al., 2013). *FTF1* resembles the *SIX* genes in terms of having mimp in its promoter region (Schmidt et al., 2013; Niño-Sánchez et al., 2016). Deletion mutants of *FTF1* and *FTF2* have implicated their role in the virulence of *Foph*; however, their functions as direct regulators of *SGE1* and *SIX* genes need further validation (Niño-Sánchez et al., 2016).

Another important transcriptional regulator in *Fom* is *FOW2* (*F. oxysporum* f. sp. *melonis* gene for wilt syndrome 2) (Imazaki et al., 2007). It is essentially required for the invasion and colonization of melon roots. Disruption of *FOW2* induced loss of virulence in *Fom*; however, it had no recognizable effect on vegetative development, conidiation, and carbon source utilization (Imazaki et al., 2007).

Expression of *SIX* genes is very low in the absence of a living plant host (Michielse et al., 2009b). Under such conditions, the activity of *SIX* genes might be suppressed by the modification of chromatin to a closed/repressive state (Schmidt et al., 2013). The repressive state is achieved by TE silencing that is guided by small RNAs transcribed from TEs. In Solanaceae members, MITEs in the vicinity of resistance genes have been shown to encode small RNAs that recruit methylation machinery to silence TEs (Kuang et al., 2009). This strategy might serve as the first layer for *SIX* gene regulation where silencing of the MITEs in the vicinity of *SIX* genes creates a closed chromatin structure (Schmidt et al., 2013). Clustering of *SIX* genes in class II TE-rich subregions of the accessory chromosome might have facilitated a coordinated expression of *SIX* genes during infection. The captured genes share the same genomic environment, i.e., closed or open chromatin structure allowing simultaneous transcriptional regulation of these genes (Schmidt et al., 2013).

### Roles of Secreted in Xylem Proteins/Genes

#### Secreted in Xylem Gene Profile Distinguishes *Formae speciales* and Races of *Fusarium oxysporum*

Fungicide treatment and soil solarization generally fail to control wilt infection in fields leaving use of resistant cultivar as the most reliable strategy of disease control (Nirmaladevi et al., 2016). Breeding of resistant cultivars requires a thorough understanding of different *formae speciales* and races of pathogen emerging in the field, which will provide timely information of genes relevant for breeding programs. Members of FOSC are devoid of discernable morphological characters and exhibit genetic heterogeneity attributed to the polyphyletic origin (Kistler, 1997) and horizontal transfer of pathogenicity-associated chromosomes (Ma et al., 2010). Discrimination between pathogenic and non-pathogenic isolates relies on pathogenicity assays that are both time consuming and strenuous owing to abundance of *formae speciales* and races in FOSC (Recorbet et al., 2003). On the other hand, standard molecular loci-based techniques used in fungal phylogenetics are also constrained by a weak correlation between pathogenicity and phylogenetic relations (Fraser-Smith et al., 2014).

**FIGURE 3:**
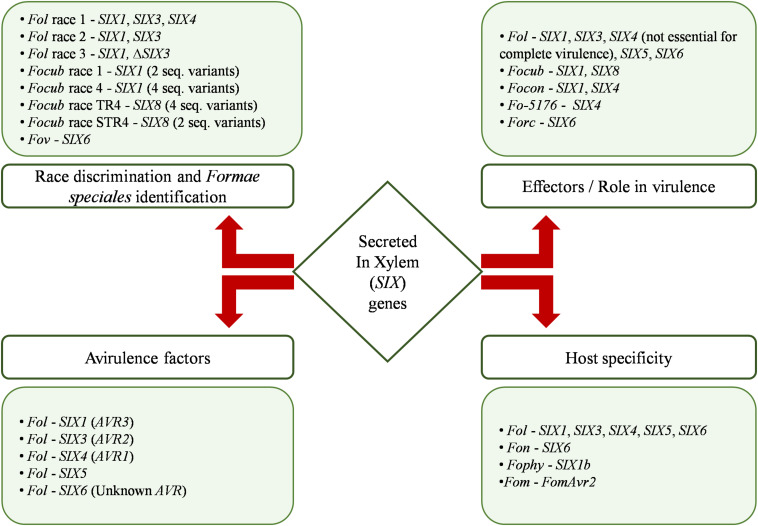
Diagrammatic summary of the roles of various Secreted in Xylem (*SIX*) genes in different *formae speciales* of *Fusarium oxysporum* species complex. *Fol*, *Fusarium oxysporum* f. sp. *lycopersici*; *Focub*, *F. oxysporum* f. sp. *cubense*; *Fov*, *F. oxysporum* f. sp. *vasinfectum*; *Focon*, *F. oxysporum* f. sp. *conglutinans*; *Fo-5176*, *Arabidopsis*-infecting strain; *Forc*, *F. oxysporum* f. sp. *radicis-cucumerinum*; *Fon*, *F. oxysporum* f. sp. *niveum*; *Fophy*, *F. oxysporum* f. sp. *physalis*; *Fom, F. oxysporum* f. sp. *melonis*; TR4, tropical race 4; STR4, subtropical race 4; AVR, avirulence gene; *SIX*, Secreted in Xylem gene.

The above challenges can be addressed by techniques that employ specific sequences of DNA closely associated with pathogenicity (Recorbet et al., 2003; van der Does and Rep, 2007; Lievens et al., 2008), such as *SIX* genes. In this regard, *SIX* genes can act as a sensitive and specific diagnostic marker as their array varies among members of different *formae speciales* and races (Lievens et al., 2008, 2009a) (diagramatically represented in Figure 3). For instance, *SIX6* gene was used as a molecular marker to differentiate cotton-specific pathogenic *Fov* isolates from non-pathogenic ones collected from the same geographical regions in Australia (Chakrabarti et al., 2011). Likewise, three races (1, 2, and 3) of pathogenic *Fol* have been distinguished on the basis of specific array and number of *SIX* genes. Isolates of race 1 show the presence of three *SIX* genes (*SIX4*, *SIX3*, and *SIX1*), while race 2 and 3 show two (*SIX3* and *SIX1*) and one (*SIX1*) genes, respectively (Rep et al., 2004; Houterman et al., 2008, 2009; Van Der Does et al., 2008; Lievens et al., 2009a; Takken and Rep, 2010; Kang et al., 2014). Furthermore, in *Focub*, race 1 (that infects Gros Michel cultivars of banana) and race 4 (pathogenic to Cavendish banana) were distinguished on the basis of presence, copy number, and sequence variability of *SIX1*. Three copies and four sequence variants were observed in race 4 compared with one copy and two variants in race 1 (Guo et al., 2014). Similarly, sequence variants of *SIX8* have been used to further differentiate race 4 into tropical (TR4) and subtropical (STR4) races. TR4 race harbors four variants, unlike two in STR4 (Fraser-Smith et al., 2014).

An account of the variation in the arsenal of *SIX* genes in various *formae speciales* reported so far is given in Supplementary Table 1. The observed variation in *SIX* gene profile among *formae speciales* can be attributed to horizontal transfer of *SIX* genes among them. Since, LS chromosome 14 carries all *SIX* genes that reside within subregions of the chromosome rich in class II TEs (Ma et al., 2010; Schmidt et al., 2013), it has been observed that a few genes or a cluster of physically linked *SIX* genes can be transferred to other strains (Simbaqueba et al., 2018). The similarity in suite of effectors and low *SIX* sequence diversity in isolates of a *forma specialis* suggest that *SIX* genes have been transferred horizontally within and among *formae speciales* of *Fo* (Lievens et al., 2009a; Fraser-Smith et al., 2014; Laurence et al., 2015; Taylor et al., 2016; van Dam et al., 2016; Czislowski et al., 2018). In a recent study, the *SIX* genes profile of strains inhabiting asymptomatic banana plants differed from the known *Focub SIX* genes (Lyons et al., 2019). Thus, a significant prospect will be to explore the differences in effector gene profile of pathogenic isolates and endophytic strains colonizing asymptomatic plants. It would aid in the accurate detection of different *formae speciales*, races, as well as endophytic strains that will be contributory in the management of diseases caused by *Fo*. Moreover, if horizontal transfer can be traced, elucidation of whether the functions of the acquired genes remain conserved in both donor and recipient *formae speciales* is necessary.

#### Virulence Function of *SIX* Genes

The presence of effector genes in *formae speciales* has been widely documented, but their function in pathogenesis has been experimentally validated only in a few (diagrammatically represented in Figure 3). The presence of *SIX1* has been reported to be a prerequisite for complete virulence of the pathogens *F. oxysporum* f. sp. *conglutinans* (*Focon*) and *Fol* on cabbage and tomato, respectively (Rep et al., 2004; Li et al., 2016). Widinugraheni et al. (2018) reported that *SIX1* homolog contributes to virulence of *Focub* tropical race 4 toward the Cavendish banana. Like *SIX1*, the role of *Fol-SIX3* in complete virulence on host plants has also been demonstrated (Van Der Does et al., 2008). The expression of *SIX1* and *SIX3* is spatiotemporally separated in *Fol*. While the expression of *Fol-SIX1* is induced in the initial phases of root colonization, *Fol-SIX3* is primarily expressed in the xylem during the later stages of hyphae growth (Van Der Does et al., 2008). Likewise, the importance of *SIX4* in virulence has been demonstrated by deletion studies in different strains. In *Focon*, deletion of *SIX4* led to a reduction in disease severity on both resistant and susceptible cabbage plants comparative to the *SIX4*-complemented and wild-type strains (Kashiwa et al., 2013). Similarly, in *Fo-5176*, *SIX4* deletion mutants exhibited reduced fungal biomass that eventually resulted in reduced disease symptoms (Thatcher et al., 2012). Deletion studies carried out in *Fol* have also highlighted the role of *SIX5* as an effector (Ma et al., 2015). *Fol-ΔSIX5* displayed an apparent reduction in disease symptoms, and reintroduction of the gene restored pathogenicity in 75% of the mutants. Furthermore, knockout mutants of *SIX6* in *Fol* and *Forc* exhibited compromised virulence confirming the role of *SIX6* in pathogenicity (Gawehns et al., 2014; van Dam et al., 2017). *SIX6* plays a role in virulence by inhibiting a hypersensitive response (HR) (Gawehns et al., 2014; De Wit, 2016). In *Nicotiana benthamiana* leaf cells (heterologous expression system), the transient expression of *Fol-SIX6*, without its signal peptide, suppressed ion leakage and cell death induced by Avr2-I-2 interaction (Gawehns et al., 2014). Like *SIX1*, *SIX8* is also required for virulence of *Focub* TR4 on Cavendish banana (An et al., 2019). Similarly, *SIX8* is involved in conferring virulence to *Fo-5176* on *Arabidopsis* and cabbage plants (Ayukawa et al., 2020).

Functional annotation is absent for most *SIX* genes. Information on how they contribute to virulence is still obscure. Some evidence suggest that they facilitate virulence by modulating hormonal pathways or defense response cascades (Thatcher et al., 2012; Ma L. et al., 2013; Gawehns et al., 2014; Ma et al., 2015). The role of the *SIX* genes in virulence, their targets, and specific biological functions of their protein products warrant more research. The protein–protein interaction assay, such as co-immunoprecipitation, pull-down assays, and yeast two-hybrid, are used to identify putative targets of effector proteins (Alfano, 2009; Rao et al., 2014). In case of transient interaction between effector and their targets, *in planta* subcellular effector localization can provide hints on target identity. Next-generation sequencing (NGS) technologies can be used to obtain sequences of putative effectors that can be screened for polymorphisms (Alfano, 2009). Identification of effector targets and information on effector polymorphisms will improve our understanding on how the pathogen triggers disease or evade recognition by the host.

#### Secreted in Xylem Genes Act as Avirulence Determinants

Recognition of effector by cognate resistance (*R*) gene product of the plant results in induction of ETI (effector-triggered immunity) in the host (Jones and Dangl, 2006). Effectors secreted by the pathogen serve as a two-edged sword (Pradhan et al., 2020). While absence of the *R* gene in susceptible plants benefits the pathogen, their presence in tolerant plants triggers innate immunity characterized by HR-mediated cell death (Flor, 1971; Jones and Dangl, 2006). However, ETI-mediated resistance to vascular wilt pathogen (*Fo*) does not include HR response; rather, it involves accumulation of tyloses, gums, phenolic compounds, and callose plugs in the xylem vessels, to preclude systemic spread of the pathogen (Mes et al., 2000; Yadeta and Thomma, 2013).

Some *SIX* gene products such as Six1, Six3, and Six4 have been found to function as avirulence determinants in tomato-*Fol* pathosystem and, correspondingly, has been termed as Avr3, Avr2, and Avr1 proteins, respectively (Rep et al., 2004; Houterman et al., 2008, 2009; Takken and Rep, 2010; Ma et al., 2015). Four resistance genes have been identified in wild tomato cultivars (*Solanum pimpinellifolium* and *Solanum pennellii*) that confer resistance against *Fol* races. These resistance genes are *I* (Immunity), *I-2*, *I-3*, and *I-7* (Bohn and Tucker, 1939; Alexander and Tucker, 1945; Stall and Walter, 1965; McGrath et al., 1987; Scott and Jones, 1989; Lim et al., 2006). Individual expression of these genes in commercial cultivars of tomato resulted in the development of resistance against race 1, race 2, and race 3. The *I* gene that encodes an LRR-RLP protein (a class of receptor-like protein) was found to provide resistance against *Fol* race 1 upon recognizing *AVR1* (Houterman et al., 2008; Catanzariti et al., 2017). Both I-3 [a cell-surface S-receptor like kinase (SRLK)] and I-7 (LRR-RLP) proteins conferred resistance against race 3 by recognizing Avr3 and Avr7, respectively (Rep et al., 2004; Lim et al., 2006, 2008; Catanzariti et al., 2015; Gonzalez-Cendales et al., 2016). Likewise, I-2 (a cytoplasmic coiled-coil nucleotide-binding leucine-rich repeat protein) provided resistance against race 2 and race 1 by recognizing Avr2 (Simons et al., 1998; Houterman et al., 2009). Later, Di et al. (2017) demonstrated that I-2 confers resistance by recognizing a specific epitope of Avr2. Ma et al. (2015) observed a new variant of the gene-for-gene model, where they observed that interaction between *SIX5* and *AVR2(SIX3)* is required for *I-2*-mediated resistance in tomato. While mutations in *SIX5* led to evasion of recognition and also compromised the virulence of *Fol*, heterologous expression of *AVR2* and *I-2* in *N. benthamiana* leaves triggered I-2-mediated cell death (Gawehns et al., 2014; Ma et al., 2015). Cao et al. (2018), using the biomolecular fluorescence complementation assay, showed that Avr2 and Six5 interact at plasmodesmata, and Six5 facilitates cell-to-cell movement of Avr2, which in I-2-containing plants results in resistance. Interestingly, Avr1 also mediates the suppression of I-triggered responses. *AVR1* gene in race 1 isolates enables the pathogen to overcome resistance response mediated by *I-2* and *I-3* despite the expression of *AVR2* and *AVR3* (Rep et al., 2005; Houterman et al., 2008). This strategy has enabled the pathogen to circumvent the emergence of new races carrying *AVR1* and *AVR3* that would retain the virulence function of *AVR3* while avoiding *I-3*-initiated resistance (Catanzariti et al., 2017). However, this suppression effect is strain-specific suggesting the involvement of an unknown fungal factor (Houterman et al., 2008; Chellappan, 2014). Suppression of resistance response by *AVR1* also established *I* as a gene of practical importance proposing that *I-3*-mediated resistance was safeguarded by deployment of *I* (Houterman et al., 2008). Nevertheless, *AVR1* was not able to overcome resistance mediated by *I-7* against race 3 (Gonzalez-Cendales et al., 2016). *I-7* is EDS1 (enhanced disease susceptibility1)-dependent and *I-2* and *I-3* are EDS1 independent (Hu et al., 2005; Gonzalez-Cendales et al., 2016). The EDS1 signaling pathway is required for basal defense and systemic-acquired resistance (Catanzariti et al., 2017). The fact that *AVR1* suppresses *I-2* and *I-3* and not *I-7* suggests that *AVR1* is not a general suppressor of basal resistance (Catanzariti et al., 2017). Recently, a new *R-AVR* interaction was recognized in melon-*Fom* pathosystem reported by Schmidt et al. (2016) wherein a novel *AVR* gene, *FomAVR2*, is recognized by *FOM2* in resistant melon plants. Similar to *SIX* genes, *FomAVR2* encodes a small secreted protein with two cysteine residues and is found associated with a mimp element in the promoter region (Schmidt et al., 2016).

Single nucleotide polymorphism serves as the source of genetic variation in *SIX* gene sequences. For instance, *SIX* genes in *Focub* were found to be present in multiple copies and showed the presence of sequence variants (Fraser-Smith et al., 2014). The gain of forms and variation in sequence of pathogenicity-associated genes are presumed as adaptations by the pathogen to respond to rapidly changing environment and host (Cuomo et al., 2007; Fraser-Smith et al., 2014; Maldonado et al., 2018). Houterman et al. (2009) demonstrated that *Fol* strains that are able to overcome *I-2*-mediated resistance carry specific point mutations in *AVR2*. These mutations in *AVR2* resulted in amino acid change in the protein that led to the loss of its avirulence function. Till now, three *AVR2* alleles have been described, each with one amino acid change at V41→M, R45→H, and R46→P in the protein (Houterman et al., 2009; Biju et al., 2017). Additionally, a race 3 isolate showed the presence of an *AVR2* gene with deletion of threonine residue at position 50 of the protein. This deletion also resulted in loss of avirulence function of Avr2 (Biju et al., 2017). Di et al. (2017) analyzed the crystal structure of one of the *AVR2* variants (*AVR2^R45H^*) that is able to evade recognition by *I-2* while retaining its virulence function. They identified two threonine residues in Avr2 protein (T53 and T145) that are required for virulence of Avr2 but not for recognition by I-2. The study revealed that the site of recognition by I-2 differs from the site required for maintenance of virulence function of Avr2. Avr2(Six3) facilitates virulence by suppression of pattern-triggered immunity (PTI) response, mainly, MAPK cascade, ROS burst, callose deposition, and growth inhibition (Di et al., 2017). Similar to *AVR2*, *AVR1*, and *AVR3* are presumed to be equally likely to undergo mutations that increase the probability of breakdown of resistance in tomato cultivars (Takken and Rep, 2010). Such studies should be extrapolated to the remaining *SIX* genes to investigate if any variation in their sequence implies structural changes in their corresponding proteins, which may potentially increase the likelihood of evasion of *SIX* gene recognition by cognate R gene. Thus, it becomes important to uncover the mode of recognition of effector proteins. Additionally, as no direct interaction of avirulence gene with the host R gene is documented in *Fol*, efforts toward mining the targets of effector and cognate R-gene proteins need more impetus to understand disease resistance in host-*Fo* pathosystems. It will also be interesting to understand how the plant’s response to other vascular wilt fungi varies from *Fo*.

Furthermore, physical linkage of certain *SIX* genes observed in various *formae speciales* is deemed as important for the functions of the interacting pair of genes. As mentioned above, the *AVR2(SIX3)–SIX5* linkage is one such example. These genes reside as a minicluster on chromosome 14 and their expression is under the control of the same bidirectional promoter present on the shared upstream region (Schmidt et al., 2013). Another pair of effector genes, *SIX8*–*PSE1*, was identified in isolates capable of infecting *Arabidopsis*, and this pair was found to be associated with suppression of resistance in *Arabidopsis* (Ayukawa et al., 2020). The mode of action of *SIX8–PSE1* potentially involves suppression of a phytoalexin called camalexin. The *SIX8*–*PSE1* pair was found to be present in head-to-head orientation similar to the *SIX3*–*SIX5* gene pair. However, unlike *SIX3*–*SIX5*, no direct interaction between *SIX8*–*PSE1* was detected in yeast two-hybrid assays. Mutation in *PSE1*, and not the *SIX8* gene, resulted in evasion of recognition by the corresponding resistance protein suggesting that *PSE1* is required to avoid detection (Ayukawa et al., 2020). A conserved gene cluster *SIX7/SIX10/SIX12* was also observed in *formae speciales narcissi* and *gladioli* (Simbaqueba et al., 2018). However, the presence of *SIX7* alone in *formae speciales cubense* and *lilii* suggests that *SIX7* may be functionally and physically separable from *SIX10* and *SIX12* (Simbaqueba et al., 2018). Clustering of *SIX* genes reflects cooperative interactions important to initiate (a)virulence functions. On this account, deletion studies either of any individual gene or a partial or a complete cluster can be done to elucidate the physical interactions among genes in a cluster and their individual roles in (a)virulence.

#### Secreted in Xylem Genes Confer Host Specificity

The capacity of fungal species to provoke disease on a specific host is referred to as host specificity. The basis of host specificity has been explained by molecular models like the guard and decoy hypothesis (Van der Biezen and Jones, 1998; Dangl and Jones, 2001; van der Hoorn and Kamoun, 2008; Zipfel and Rathjen, 2008). These models have tremendously contributed in deciphering the role of effectors on the virulence of the pathogen as well as understanding the underpinnings of host–pathogen interactions (van der Hoorn and Kamoun, 2008; Borah et al., 2018).

It is widely accepted that the factors that contribute to (a)virulence of a pathogen also determine its host specificity (Li et al., 2021). In this regard, there are studies where *SIX* genes have been implicated in imparting host specificity to the pathogen. Avirulence genes of *Fol* and *Fom* that confer resistance to races of tomato and melon, respectively, function as host-specific factors (Rep et al., 2004; Houterman et al., 2008, 2009; Catanzariti et al., 2015; Schmidt et al., 2016). *SIX6* gene from *Fon* has been known to operate as an avirulence gene in watermelon-*Fon* pathosystem providing host specificity to the pathogen (Niu et al., 2016). The role of *F. oxysporum* f. sp. *physalis* (*Fophy*) *SIX1* gene in specificity was demonstrated through complementation experiment where complementation strains of two homologs of *Fophy-SIX1* (a and b) failed to overcome virulence loss in *Fol-ΔSIX1* transformants (Simbaqueba et al., 2018). Interestingly, *SIX1b* complementation restored avirulence of *Fol* on IL7-3 transgenic tomato lines carrying *I-3*, demonstrating that *Fophy-SIX1b* is recognized by the resistance gene and functions as an avirulence factor. Similarly, complementation of *Focon*–Δ*SIX1* mutant using *Fol*–*SIX1* failed to rescue the virulence of *Focon* on cabbage suggesting a host-specific role of *Fol*–*SIX1* (Li et al., 2016).

*Formae speciales* of *Fo* are generally host specific but *Forc* shows an exceptional host range. *Forc* infects cucumber, melon, watermelon, squash, and gourd (Vakalounakis, 1996; Punja and Parker, 2000; Cohen et al., 2015). Previous studies have demonstrated that *forma specialis cucumerinum* showed mild cross-pathogenicity toward melons (Cafri et al., 2005) that was later corroborated in the findings of a study by van Dam et al. (2016). They assessed cross-pathogenicity of cucurbit-infecting strains (*cucumerinum*, *radicis-cucumerinum*, *melonis*, and *niveum*) on resistant and susceptible cultivars of their corresponding hosts. The study revealed that *Fom* and *Fon* were highly host specific, whereas isolates of *Forc* displayed some degree of cross-pathogenicity toward musk melon (van Dam et al., 2016). The genetic mechanism underlying the difference in host range was examined, and results showed that effector genes present on the mobile pathogenicity chromosome of *Forc* and *Fom* limit host range (van Dam et al., 2017). A close comparison of the mobile pathogenicity chromosomes of *Forc* and *Fom* revealed that a single gene on mobile pathogenicity chromosome of *Fom* determined this difference in host range. This gene, upon introduction in *Forc*, rendered it non-pathogenic on cucumber suggesting that the gene functions as an avirulence factor (Li et al., 2021). Overall, studies devoted to discerning the role of *SIX* genes in determining host-specificity in members of FOSC are sparse (Table 1), obstructing our understanding of host specialization. Hence, more studies are required to identify gene(s) involved in limiting the host range of FOSC members.

**TABLE 1:** Secreted in Xylem genes in *formae speciale*s of *Fusarium oxysporum*, their role in (a) virulence and host specificity.

*F. oxysporum formae speciales*	*SIX* gene	Role in virulence	Role in host specificity	Avirulence gene	Resistance gene	Type of resistance gene	Source of resistance gene	References
*Arabidopsis-infecting Fo-5176*	*SIX4*	Yes	NK	NK	NK	NK	NK	Thatcher et al., 2012
	*SIX8*	Yes	NK	NK	NK	NK	NK	Ayukawa et al., 2020
*lycopersici*	*SIX1*	Yes	Yes	*Avr3*	*I-3*	SRLK	Wild tomato *Solanum pennellii*	Scott and Jones, 1989; Rep et al., 2004; Catanzariti et al., 2015
	*SIX3*	Yes	Yes	*Avr2*	*I-2*	CC-NB-LRR	Wild tomato *S. pimpinellifolium*	Stall and Walter, 1965; Simons et al., 1998; Houterman et al., 2008, 2009; Ma et al., 2015
	*SIX4*	No	Yes	*Avr1*	*I*	LRR-RLP	Wild tomato *S. pimpinellifolium*	Bohn and Tucker, 1939; Houterman et al., 2008; Thatcher et al., 2012; Catanzariti et al., 2017
	*SIX5*	Yes	Yes	NK	NK	NK	NK	Ma et al., 2015
	*SIX6*	Yes	NK	NK	NK	NK	NK	Gawehns et al., 2014
	NK	NK	Yes	*Avr7*	*I-7*	LRR-RLP	Wild tomato *S. pennellii*	McGrath et al., 1987; Lim et al., 2006; Gonzalez-Cendales et al., 2016
*conglutinans*	*SIX1*	Yes	NK	NK	NK	NK	NK	Li et al., 2016
	*SIX4*	Yes	NK	NK	NK	NK	NK	Kashiwa et al., 2013
*cubense*	*SIX1*	Yes	MK	NK	NK	NK	NK	Widinugraheni et al., 2018
	*SIX8*	Yes	NK	NK	NK	NK	NK	An et al., 2019
*melonis*	NK	NK	Yes	*Fom- g14035*	NK	NK	NK	Li et al., 2021
	NK	Yes	Yes	*FomAVR2*	*Fom*-2	NB-LRR	Melon cultivar CH17187	Joobeur et al., 2004; Schmidt et al., 2016
*niveum*	*SIX6*	Yes	Yes	NK	NK	NK	NK	Niu et al., 2016
*physalis*	*SIX1b*	Yes	Yes	NK	NK	NK	NK	Simbaqueba et al., 2018
*radicis-cucumerinum*	*SIX6*	Yes	NK	NK	NK	NK	NK	van Dam et al., 2017

*SIX, Secreted in Xylem; NK, not known; Avr, avirulence gene; I, immunity gene; SRLK, S-receptor-like kinase; CC-NB-LRR, coiled-coil nucleotide-binding leucine-rich repeat; LRR-RLP, leucine-rich repeat receptor-like protein; NB-LRR, nucleotide-binding leucine-rich repeat.*

## Conclusion

Even though *F. oxysporum* infects a variety of plant species, the investigations on the molecular basis of pathogenicity are restricted to a limited number of hosts, mainly tomato, banana, melons, cucurbits, and cabbage. Genome sequencing of tomato and other cucurbit-infecting pathogens has provided insights on host–pathogen interactions, but a large number of *formae speciales* are yet left unexplored. Genome-based approaches are needed to elucidate mechanisms and understand the evolution of the pathogenicity. Transcriptomic and proteomic studies in infected plant tissues, the role of transposons and HGT in genome structure modulation, and emergence of host-specific pathogenicity are particular areas of interest. A significant prospect is to explore the differences in effector gene profile between different *formae speciales*, races, and non-pathogenic isolates. It would aid in the molecular detection of different *formae speciales* and pathogenic races that will assist significantly in the management of diseases caused by *Fo*. In addition, the biological functions of effector genes are still under investigation and require exhaustive research.

## Author Contributions

PJ and RK jointly conceptualized and wrote the manuscript, contributing major parts of the literature survey. All the authors have collectively reviewed the manuscript and approved it.

## Conflict of Interest

The authors declare that the research was conducted in the absence of any commercial or financial relationships that could be construed as a potential conflict of interest.
