# Supplementary material for: Secreted in Xylem Genes: Drivers of Host Adaptation in Fusarium oxysporum
Source: Front Plant Sci. 2021 Apr 22;12:628611. doi: 10.3389/fpls.2021.628611 (PMC8101498; doi:10.3389/fpls.2021.628611)
Supplement: Supplementary file 1 [file Table_1.docx]

| **Supplementary Table 1: Distribution of Secreted in Xylem genes in members of  *Fusarium oxysporum* species complex** | | | | | | | | | | | | | | | | |
| --- | --- | --- | --- | --- | --- | --- | --- | --- | --- | --- | --- | --- | --- | --- | --- | --- |
| ***F. oxysporum formae* *speciales***  **(f. sp.)** | **Host** | ***SIX* Genes** | | | | | | | | | | | | | | **Reference*** |
|  |  | **1** | **2** | **3** | **4** | **5** | **6** | **7** | **8** | **9** | **10** | **11** | **12** | **13** | **14** |  |
| *Arabidopsis-* infecting *(Fo-5176)* | *Arabidopsis,*  *Brassica* spp. | + | – | – | + | – | – | – | + | + | – | – | – | – | – | Thatcher et al., 2012  Czislowski et al., 2018  Williams et al., 2016  van Dam et al., 2017 |
| *apii* | *Apium* sp*.* | + | – | – | – | – | – | – | – | – | – | – | – | – | – | Henry et al., 2020 |
| *betae* | *Beta* *vulgaris* | + | – | – | – | – | + | – | – | – | – | – | – | – | – | Covey et al., 2014 |
| *canariensis* | *Phoenix* spp. | + | – | – | – | – | – | + | – | – | + | – | + | – | – | Laurence et al., 2015  Ponukumati et al., 2019 |
| *cepae* | *Allium* *sativum* | – | – | + | – | + | – | + | – | + | + | – | + | – | + | Sasaki et al., 2015  Taylor et al., 2016  Armitage et al., 2018 |
| *ciceris* | *Cicer arietinum* | – | – | – | – | + | – | – | + | – | – | + | – | + | + | Williams et al., 2016 |
| *conglutinans* | *Brassica oleracea* | + | – | – | + | – | – | – | + | – | – | – | – | – | – | Li et al., 2016  Kashiwa et al., 2013  Czislowski et al., 2018 |
| *coriandrii* | *Coriandrum* sp*.* | + | – | – | – | – | – | – | – | – | – | – | – | – | – | Henry et al., 2020 |
| *cubense* | *Musa* spp. | + | + | – | + | – | + | + | + | + | + | – | – | + | – | Meldrum et al., 2012  Fraser-smith et al., 2014  Guo et al., 2014  Widinugraheni et al., ‎2018  Czislowski et al., 2018  Williams et al., 2016  van Dam et al., 2016 |
| *cucumerinum* | *Cucumis* spp. | – | – | – | – | – | + | – | – | – | – | – | – | – | – | Rocha et al., 2016  van Dam et al., 2016 |
| *dianthi* | *Dianthus* *caryophyllus* | – | – | – | – | – | – | + | – | + | + | – | + | – | – | Taylor et al., 2016 |
| *elaeidis* | *Elaeis guineensis* | – | – | – | – | – | – | – | + | + | – | + | – | – | – | Adusei-fosu and Dickinson, 2019 |
| *fragariae* | *Fragaria* spp. | _+_ | – | – | – | – | – | – | – | – | – | – | – | + | – | Czislowski et al., 2018 |
| *freesia* | *Fragaria* spp. | – | – | – | – | – | – | + | – | – | + | – | + | + | + | Taylor et al., 2016 |
| *Human-*infecting | *Homo sapiens* | – | – | – | – | – | – | – | + | – | – | – | – | – | – | Czislowski et al., 2018 |
| *koae* | *Acacia* sp. | + | – | – | – | – | + | – | – | – | – | – | – | – | – | Dobbs et al., 2020 |
| *lilii* | *Lilium* spp. | – | – | – | – | – | – | + | – | – | – | – | – | – | – | Lievens et al., 2009b |
| *lini* | *Linum* spp. | + | – | – | – | – | – | + | – | – | + | – | + | + | – | Laurence et al., 2015  Taylor et al., 2016 |
| *luffae* | *Luffa aegyptiaca* | – | – | – | – | – | + | – | – | – | – | – | – | – | – | van Dam and Rep, 2017 |
| *lycopersici* | *Solanum lycopersicum* | + | + | + | + |  | + | + | + | + | + | + | + | + | + | Rep et al., 2004  Czislowski et al., 2018  Houterman et al., 2007  Lievens et al., 2009b  Carmona et al., 2020  van Der Does et al., 2008 |
| *medicaginis* | *Medicago* *sativa* | + | – | – | – | – | – | – | + | + | – | – | – | + | – | Rocha et al., 2016  Williams et al., 2016  Thatcher et al., 2016  Czislowski et al., 2018 |
| *melonis* | *Cucumis* *sativus* | + | – | – | – | + | + | – | + | + | – | + | – | + | – | van Dam et al., 2016, 2017  Lievens et al., 2009b  Czislowski et al., 2018  Williams et al., 2016 |
| *momordicae* | *Momordica charantia* | + | – | – | – | – | + | – | – | – | – | – | – | – | – | van Dam and Rep, 2017 |
| *narcissi* | *Narcissus* spp. | – | – | – | – | – | – | + | – | + | + | – | + | + | – | Taylor et al., 2016, 2019 |
| *niveum* | *Citrullus* *lanatus* | + | – | – | + | – | + | – | + | + | – | + | – | + | – | Meldrum et al., 2012  Niu et al., 2016 Charkrabarti et al., 2011  Taylor et al., 2016  van Dam et al., 2016  Czislowski et al., 2018 |
| *palmarum* | *Washingtonia robusta* | – | – | – | – | – | – | – | + | + | + | – | – | – | – | Ponukumati et al., 2019 |
| *passiflorae* | *Passiflora* *edulis* | – | – | – | – | – | + | – | + | + | – | + | – | – | – | Rocha et al., 2016  Charkrabarti et al., 2011  Gawehns et al., 2014  Czislowski et al., 2018 |
| *phaseoli* | *Phaseolus* sp. | + | – | – | – | – | + | – | + | – | – | + | – | – | – | Taylor et al., 2016  Nino-Sanchez et al., 2015 |
| *physali* | *Physalis peruviana* | + | – | – | – | – | – | + | – | – | + | – | + | – | – | Simbaquea et al., 2018 |
| *pisi* | *Pisum* *sativum* | + | – | – | – | – | + | + | – | + | + | + | + | + | + | Czislowski et al., 2018  Chakrabarti et al., 2011  Lievens et al., 2009b  Meldrum et al., 2012  Thatcher et al., 2012  Guo et al., 2014  Rocha et al., 2016  Williams et al., 2016  Taylor et al., 2016 |
| *radicis*–*cucumerinum* | Root rot in *Cucumis* spp. | – | – | – | – | – | + | – | – | + | – | + | – | + | – | Lievens et al., 2009b  Chakrabarti et al., 2011  van Dam et al., 2017 |
| *raphani* | *Raphanus sativus* | – | – | – | – | – | – | – | + | + | – | – | – | – | – | Czislowski et al., 2018 |
| *spinaceae* | *Spinacea oleracea* | – | – | – | + | – | – | – | + | + | – | – | – | – | – | Batson et al., 2020 |
| *sesame* | *Sesamum indicum* | + | – | + | – | – | – | + | + | + | + | + | – | + | + | Duan et al., 2020 |
| *vasinfectum* | *Gossypium* spp. | – | – | – | – | – | + | – | – | + | – | – | – | + | – | Chakrabarti et al., 2011  Czislowski et al., 2018 |
| *zingiberi* | *Zingiber* *officinale* | – | – | – | – | – | – | + | – | + | + | – | + | – | – | Czislowski et al., 2018 |
| *SIX,* Secreted in xylem;*+,* Documented; *–,* Not documented  *****References for these citations can be found the Reference list in the main article: https://doi.org/10.3389/fpls.2021.628611 | | | | | | | | | | | | | | | | |
